# Publication of Decision Model Source Code: Attitudes of Health Economics Authors

**DOI:** 10.1007/s40273-019-00796-3

**Published:** 2019-05-08

**Authors:** Joanna Emerson, Rachel Bacon, Alma Kent, Peter J. Neumann, Joshua T. Cohen

**Affiliations:** 1 Center for the Evaluation of Value and Risk in Health, Institute for Clinical Research and Health Policy Studies, Tufts Medical Center, Boston, MA 02111, USA

Dear Editor,

Computer decision models serve as the analytic foundation for most health economic evaluations, the prominence of which continues to grow. Cohen et al. [1], Cohen and Wong [2], and Sampson and Wrightson [3] have argued that authors should publish the decision model ‘source code’, by which we mean the model’s human-readable computer instructions or its underlying component files with formulas in the case of spreadsheet-implemented models. They explained that releasing source code would boost model credibility and allow other researchers to adapt existing computer code to answer similar and related questions, thus increasing the efficiency of health economics. Others (e.g., Padula et al. [4]) have argued against publication of source code, citing intellectual property concerns and the potential for models to be misused to promote misleading claims. We conducted a survey of health economic article authors regarding their willingness to publicly release their model source code.

The Center for the Evaluation of Value and Risk in Health (CEVR) at Tufts Medical Center (Boston, MA, USA) has developed an online ‘Open-Source Model Clearinghouse’. Funded by the Bill and Melinda Gates Foundation, the clearinghouse encourages investigators to publicly post their models, and helps others to locate those models and download the code.

As the clearinghouse website (http://www.GHCEARegistry.org) explains, CEVR asks each author to provide summary information about their model (e.g., disease or condition modeled, intervention, etc.) and limits clearinghouse contents to “computer simulations developed to support original economic assessments of health interventions.” CEVR does “not evaluate the correctness or quality of posted models,” but instead aims to “facilitate ‘crowdsourcing’ of model review by individuals interested in each particular model’s problem area.” Nor does CEVR “require model documentation,” as we aim to allow the health economics community to establish de facto documentation standards that balance the need for adequate clarity with the amount of work attending model publication.

We emailed primary authors of articles describing original cost per disability-adjusted life-year (DALY) averted analyses, published in peer-reviewed journals from 2010 to 2017. The Tufts Global Health CEA Registry (www.GHCEARegistry.org) catalogs all such articles published in English. We targeted cost-per-DALY article authors for this survey because the open-source registry work is supported by the Bill and Melinda Gates Foundation, which is also supporting our cataloging of the cost-per-DALY literature, and which has an interest in promoting the sharing of information most salient to low and middle-income countries.

We asked authors if they would post their code (or executable models) in the clearinghouse. We sent an initial email and one reminder email between August 8, 2018 and September 6, 2018. We invited only those authors answering in the affirmative to our initial survey to follow up by actually posting their models. We asked authors who said they would not post their code to identify concerns that factored into their decision.

We sent emails to 337 authors and received 89 bounce-back messages, resulting in a final sample of 248 distinct authors. We received 18 responses (7.3%). Five authors agreed to post their code in the clearinghouse. Of the five, four ultimately submitted models. The other author in this group declined to post code, stating that their model “needs to be refined further before publication.”

The 13 responding authors who declined to post their code selected the following reasons for their decision (responses not mutually exclusive): need to document code (6), need to improve code before release (3), and intellectual property concerns (3). Authors could also provide open-text responses; those we received expressed concerns regarding intellectual property, the effort needed to document code and provide technical assistance, and the potential for models to be misused.

Because our response rate was only 7.3%, we cannot say that our results reliably characterize the factors that typically discourage authors from publishing their model source code. However, the low response rate itself might be considered a telling finding. Beyond revealing that health economists are busy people who do not relish responding to unsolicited surveys, it suggests that many authors may not want to confront the issue of publishing their source code, or at the very least, may not view source code publication as a priority.

Perhaps most of all, the findings suggest that there simply is no cultural expectation that models should, when possible or to the extent possible, be made public. Given that public release of code requires effort (despite our intention to minimize that burden), involves the risk of opening one’s work to criticism, and sacrifices some intellectual property prerogatives, authors mostly ignored our request.

The question is—How might this calculus be changed? The main levers, we believe, are sponsors and journals. The pharmaceutical industry sponsors a sizeable portion of the work in the field, for example. Nearly one-third of the cost-effectiveness articles published from 2000 to 2012 and catalogued in the Tufts CEA Registry (our repository of over 7000 cost per quality-adjusted life-year studies) report industry sponsorship [5]. Corporations cannot fully release some models because doing so might reveal confidential patient or business information, or may constitute communication of scientific claims for which the evidence does not satisfy regulatory criteria. Even in these cases, however, we believe that it is possible to develop model versions that make the most of the underlying assumptions open to inspection while protecting information that must be kept confidential. Other influential organizations, including government bodies, such as the National Institute for Health and Care Excellence (NICE) in England, or nonprofit entities, such as the Institute for Clinical and Economic Review (ICER) in the US, could perhaps require open publication of models provided by contractors or other parties [6]. Journals may fear that if they impose release requirements, authors will instead turn to other journals. However, the fact that some high-profile journals have implemented open data standards [7] suggests that there is a way forward. Certainly, the most prestigious journals—those with the highest impact factor ratings—could lead the way without fear that authors would shun them. Other journals could follow.

Ultimately, it is up to authors to foster a new set of cultural expectations. As we have argued elsewhere, “Open model publication bolsters credibility by allowing others to assess independently whether alternative, plausible assumptions substantially alter model projections” [2]. Without allowing that kind of inspection, we will continue the reality that our field rests largely on faith rather than the principles of scientific exchange, which means reproducibility of results and the presentation and open challenge of ideas. Under these circumstances, “health economics will have—and indeed should have—limited influence on individual but especially societal health policy decisions” [1]. We are hopeful that the health economics community will move towards more openness, and as a result, decision makers will be comfortable using the insights to be gained from our field’s systematic assessment of the costs and gains attending alternative investments in public health.

You can find our full survey and more detailed results at: https://osf.io/n9xzp/.
