# Correction to: Publication of Decision Model Source Code: Attitudes of Health Economics Authors

**DOI:** 10.1007/s40273-019-00813-5

**Published:** 2019-06-19

**Authors:** Joanna Emerson, Rachel Bacon, Alma Kent, Peter J. Neumann, Joshua T. Cohen

**Affiliations:** Center for the Evaluation of Value and Risk in Health, Institute for Clinical Research and Health Policy Studies, Tufts Medical Center, Boston, MA 02111 USA

## Correction to: PharmacoEconomics 10.1007/s40273-019-00796-3

The article Publication of Decision Model Source Code: Attitudes of Health Economics Authors written by Joanna Emerson, Rachel Bacon, Alma Kent, Peter J. Neumann, Joshua T. Cohen was originally published electronically on the publisher’s internet portal (currently Springerlink) on May 8, 2019 without open access.

With the author(s)' decision to opt for Open Choice the copyright of the article changed on June 2019 to © The Author(s) (2019) and the article is forthwith distributed under the terms of copyright.

The original article was corrected.

